# Integrated knowledge translation to advance noncommunicable disease policy and practice in South Africa: application of the Exploration, Preparation, Implementation, and Sustainment (EPIS) framework

**DOI:** 10.1186/s12961-021-00733-x

**Published:** 2021-05-17

**Authors:** Nasreen S. Jessani, Anke Rohwer, Bey-Marrie Schmidt, Peter Delobelle

**Affiliations:** 1 Division of Epidemiology and Biostatistics, Centre for Evidence-Based Health Care, Faculty of Medicine and Health Sciences, Stellenbosch University, Cape Town, South Africa; 2 Department of International Health, Johns Hopkins Bloomberg School of Public Health, Baltimore, USA; 3 Cochrane South Africa, South African Medical Research Council, Cape Town, South Africa; 4 School of Public Health, University of the Western Cape, Cape Town, South Africa; 5 Chronic Disease Initiative for Africa, University of Cape Town, Cape Town, South Africa; 6 Department of Public Health, Vrije Universiteit Brussel, Brussels, Belgium

**Keywords:** South Africa, Low- and middle-income countries, Integrated knowledge translation, Research uptake, Embedded research, Implementation research, Noncommunicable diseases, Stakeholder engagement, Exploration, preparation, implementation, and sustainment framework, Evidence-informed decision-making

## Abstract

**Background:**

In response to the “know–do” gap, several initiatives have been implemented to enhance evidence-informed decision-making (EIDM). These include individual training, organizational culture change management, and legislative changes. The importance of relationships and stakeholder engagement in EIDM has led to an evolution of models and approaches including integrated knowledge translation (IKT). IKT has emerged as a key strategy for ensuring that engagement is equitable, demand-driven, and responsive. As a result, the African-German Collaboration for Evidence-Based Healthcare and Public Health in Africa (CEBHA+) incorporated an IKT approach to influence noncommunicable diseases (NCD) policy and practice. We documented the phased process of developing, implementing, and monitoring the IKT approach in South Africa; and explored the appropriateness of using the exploration, preparation, implementation, and sustainment (EPIS) framework for this purpose.

**Methods:**

We mapped the South Africa IKT approach onto the EPIS framework using a framework analysis approach. Notes of team meetings, stakeholder matrices, and engagement strategies were analysed and purposefully plotted against the four phases of the framework in order to populate the different constructs. We discussed and finalized the analysis in a series of online iterations until consensus was reached.

**Results:**

The mapping exercise revealed an IKT approach that was much more iterative, dynamic, and engaging than initially thought. Several constructs (phase-agnostic) remained important and stable across EPIS phases: stable and supportive funding; committed and competent leadership; skilled and dedicated IKT champions; diverse and established personal networks; a conducive and enabling policy environment; and boundary-spanning intermediaries. Constructs such as “innovations” constantly evolved and adapted to the changing inner and outer contexts (phase-specific).

**Conclusions:**

Using the EPIS framework to interrogate, reflect on, and document our IKT experiences proved extremely relevant and useful. Phase-agnostic constructs proved critical to ensure resilience and agility of NCD deliberations and policies in the face of highly dynamic and changing local contexts, particularly in view of the current coronavirus disease 2019 (COVID-19) pandemic. Bridging IKT with a framework from implementation science helps to reflect on this process and can guide the development and planning of similar interventions and strategies.

## Background

Effective translation of research evidence into policy and practice is important for improving health outcomes and reducing health inequities [1, 2]. There are several complexities, however, associated with how and to what extent research evidence is translated into policy and practice [2]. These complexities (also referred to as the “know–do” gap) can be explained by factors related to researchers, decision-makers, their engagement, and the context in which they operate [3–7]. For example, researchers may misunderstand the needs of decision-makers and present the “right answers to the wrong questions” [8]. Decision-makers may not use existing research evidence in their decision-making, or they may use research evidence in a manner that was not intended by the research community, or they may adapt research evidence to fit certain political needs and agendas [8]. Similarly, decision-makers are often unable to articulate their research needs as answerable research questions [2, 9, 10]. Continuous and effective engagement between researchers and decision-makers requires time and resources. It is often reliant on long-standing and/or personal relationships, which has advantages, such as long-term trust and confidence in the partnership, but also risks when key actors for example change position and are no longer able to facilitate the engagement [11, 12]. Complex social and political factors (played out in the relationship between researchers and decision-makers) also enhance or hinder effective use or uptake of research evidence into policy and practice [13].

In response to the “know–do” gap, several initiatives have been promoted, including individual training, organizational culture change management, and legislative changes such as resolutions to better use research evidence for decision-making [14]. This has given rise to several models and frameworks for enhancing evidence-informed decision-making (EIDM) [15–21]. The increased focus on the importance of relationships [11, 12, 22–26] and stakeholder engagement [8, 27–31] led to further evolution of these frameworks to what is now referred to as integrated knowledge translation (IKT): “an approach or set of processes that can lead to the generation of knowledge for optimizing healthcare delivery systems and improving health system performance and associated outcomes” [32]. Implementation science, on the other hand, is defined as the “scientific study of methods to promote the systematic uptake of research findings and other evidence-based practices into routine practice, and, hence, to improve the quality and effectiveness of health services and care” [33]. Applying an implementation science lens to systematically reflect on and draw out experiences of IKT approaches or processes provides an opportunity to address the “know–do” gap [34]. Additionally, there is a need for research on the implementation, monitoring and evaluation of interventions or approaches for translating research evidence in health policy and practice decision-making, specifically in African settings [35, 36].

The Collaboration for Evidence-Based Healthcare and Public Health in Africa (CEBHA+) (https://www.cebha-plus.org/) is an African-German research consortium funded by the Federal Ministry of Education and Research (BMBF) in Germany. It focuses on three noncommunicable diseases (NCDs) (road traffic injuries, diabetes, and hypertension), and includes an IKT approach to ensure the uptake and use of research. Research objectives for each partner country were formulated as part of a priority-setting exercise carried out with health decision-makers and researchers in sub-Saharan Africa [37]. The five African country partners (Ethiopia, Malawi, Rwanda, Uganda, and South Africa) developed country-specific IKT approaches, which are currently implemented and monitored alongside the different research activities. In South Africa, the CEBHA+ country partners include the Centre for Evidence-Based Health Care (CEBHC) at Stellenbosch University, the Chronic Disease Initiative for Africa (CDIA) at the University of Cape Town, and Cochrane South Africa (CSA) at the South African Medical Research Council (SAMRC).

**Fig. 1:**
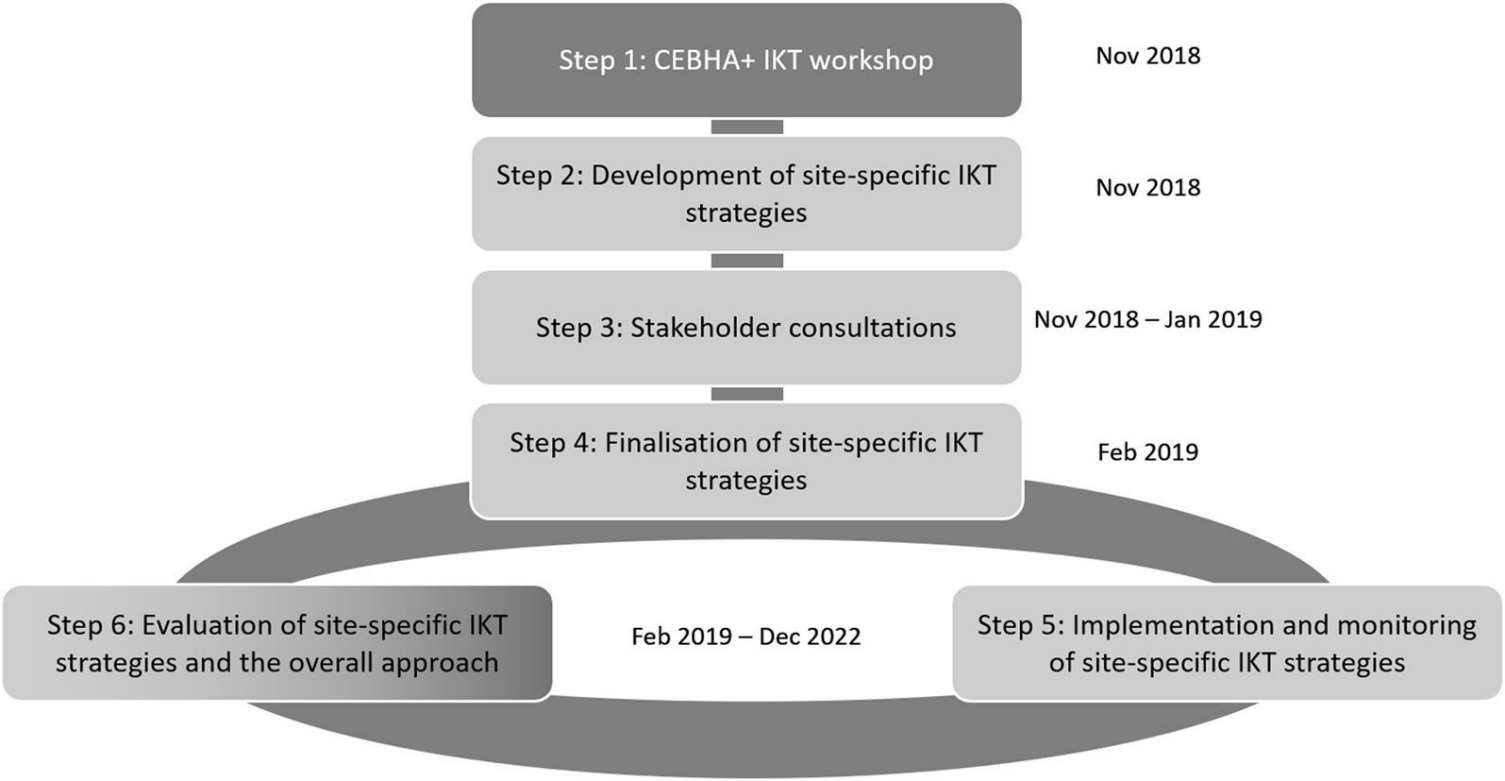
Planned IKT approach [38]

The CEBHA+ coordinated IKT approach was envisioned as a six-step process [38] (Fig. 1) that commenced with a foundational workshop for all CEBHA+ partners. This workshop was designed and implemented by CEBHC [39] in October 2018. Draft stakeholder analyses and IKT strategies for key identified CEBHA+ stakeholders were developed by each CEBHA+ African country at the workshop (Step 1). These were further refined with input from objective colleagues (Step 2) several of the identified stakeholders (Step 3), and then finalized (Step 4). While the strategies varied by stakeholder and country, in South Africa some of the activities included regular scheduled meetings between CEBHA+ and stakeholders; being available for ad hoc consultations on NCD-related matters; tailoring outputs to their preferences; organizing a national NCD symposium; disseminating research products such as publications, presentations, and issue briefs; and providing regular programme updates. Implementation commenced in February 2019, with monitoring embedded throughout the process (Step 5) and an overall evaluation of IKT activities and processes across the consortium (Step 6). The entire process, however, was iterative in nature and supported by a working group with IKT methods expertise, training opportunities. Step 6—a semi-external cross-country evaluation of the CEBHA+ IKT approach—will be implemented by one of the German partners, the Ludwig Maximilian University of Munich (LMU), which is not directly involved in operationalizing country-specific IKT approaches [38].

The objective of this paper is to complement the multi-site semi-external evaluation with a deep-dive description of the South African six-step IKT approach, using an implementation science lens guided by the exploration, preparation, implementation, and sustainment (EPIS) framework [40]. This will lead to two outcomes: one, a documentation of the phased process of developing, implementing and monitoring the IKT approach, in order to contribute to growing research in the field from African settings; and two, exploration of the appropriateness and relevance of the EPIS framework for this purpose, thereby providing new insights into the intersection between IKT and implementation science.

## Methods

We used a systematic approach to reflect on and map the planning and implementation of our IKT approach based on a cycle of internal discussions, including exploring the relevance of existing implementation frameworks. The discussion was initiated by a group deliberation in workshop format to screen the constructs of the Consolidated Framework for Intervention Research (CFIR) developed by Damschroder et al. [41] for its applicability to mapping the IKT approach in South Africa. CFIR is a well-known and popular framework that presents a taxonomy for conceptualizing and distinguishing between a wide range of contextual determinants of implementation success, including characteristics of the intervention, the implementing organization (inner setting), the outer setting, characteristics of individuals, and details of the implementation process. Given that deductive reasoning can be used to identify barriers and enablers that influence implementation outcomes, CFIR is considered a “determinants” framework in the classification of models, theories, and frameworks of implementation science proposed by Nilsen [42].

Despite its comprehensiveness and flexibility, however, the CFIR was found to be less suitable to capture the dynamics at play between the inner and outer settings of IKT implementation in our context [43], which involved boundary spanners and the importance of reciprocity in stakeholder relationships. It was decided to revisit the literature and conduct a more extensive mapping of available implementation frameworks and constructs, informed by the review by Tabak et al. [44] and complemented by models included in the Dissemination-Implementation Webtool (https://dissemination-implementation.org/content/diMain.aspx) based on a consensus to identify a model that would capture the stages of operationalization from conception to sustainability; interconnections between people, institutions, and constructs; and knowledge translation models used in health services policy. Several models were assessed based on their field of origin (public/health services sector), construct flexibility, implementation focus, and application at different levels of the socio-ecological framework [40, 45–48] and the EPIS framework selected for this paper. Table 1 below provides a summary of the models that were assessed, as well as their relative applicability to this study.

**Table 1:** Dissemination and implementation models and frameworks assessed

Model/framework	D/I^#^	Description	Pros	Cons
Research Development Dissemination and Utilization Framework [49]	D = I	Model of social system of knowledge transfer focused on understanding processes of innovation, dissemination, and knowledge utilization	• Focus on 4 socio-ecological levels: individual, interpersonal, organizational, and (social) system level • Stresses the importance of a “linkage” model for describing collaborative interaction between user and resource systems related to internal and external resources • Focus on research utilization	•Oriented towards dissemination and utilization in social science/education • Focus on traditional knowledge translation (i.e. research utilization and dissemination only) • Does not include the policy socio-ecological level •Low construct flexibility • No figure associated with this model
Conceptual Model of Knowledge Utilization [50, 51]	D	Model for knowledge utilization among United States state agency officials focusing on contextual, technical, and bureaucratic variables	• Focus on 3 socio-ecological levels: community, system, and policy socio-ecological levels • Focus on knowledge utilization in public policy • Highlights the importance of contextual variables	• Dissemination only • Focus on knowledge utilization • Does not include individual or organizational socio-ecological levels
Research Knowledge Infrastructure [19]	D > I	Framework for knowledge translation based on five elements (message, target audience, messenger, KT processes, and evaluation)	• Good construct flexibility • Focus on 4 socio-ecological levels: individual, community, organization, and policy level	• Lacks systems focus • Generic knowledge transfer framework • No figure associated with this model
Promoting Action on Research Implementation in Health Services (PARIHS) [52]	I	Multidimensional evidence-based healthcare *determinant* framework that focuses on the dynamic relationship among evidence, context, and facilitation	• Focus on 3 socio-ecological levels: individual, organizational, community • Refined framework focusing on integration of theoretical concepts and diagnostic and evaluative measures • Good conceptual integrity, and face and concept validity	• Developed for use in evidence-based healthcare in nursing practice • No focus on systems/policy level
Pathways to Evidence-Informed Policy [53]	I > D	Policy and practice framework outlining three stages (adopt, adapt, and act) in the uptake of evidence	• Focuses on 3 socio-ecological levels: individual-, organizational- and systems-level factors in the decision-making process • Emphasizes the policy context and its influence on each stage of the interaction between research, evidence, and the policy process	• Conceptualized as a pathway rather than framework • Focuses on policy ideas as the starting point for health decision-making • No figure associated with this model
Consolidated Framework for Implementation Research (CFIR) [41]	I	Meta-theoretical *determinants* framework which identifies constructs across five domains (intervention, inner and outer setting, characteristics of the individual, and process)	• Focus on 5 socio-ecological levels: individual, organization, community, systems, and policy level • Highly cited framework which offers an overarching typology to promote implementation • Complements process and evaluation theories • Specifically developed for the field of health services	• Taxonomy of dissemination and implementation (D&I) constructs rather than operational framework • Lacks focus on dynamic and interlinking factors
Exploration, Preparation, Implementation, Sustainment (EPIS) framework [34, 54]	I	Framework with four distinct phases to guide and describe implementation (exploration, preparation, implementation, sustainment) that outlines the factors that bridge the outer and inner context, as well as the interconnections and interlinkages that characterize the dynamics, complexity, and interplay of contexts	• Focus on 5 socio-ecological levels: individual, organization, community, systems, and policy level • Offers a temporal element against which to plot D&I constructs • Research-oriented	• Developed for public services in general • Limited prescriptive guidance for its use
Evidence Integration Triangle [46]	I > D	Three-pronged model which emphasizes the interaction between evidence-based interventions, longitudinal measures of progress, and participatory implementation processes	• Focus on 5 socio-ecological levels: individual, organization, community, system, and policy levels • Centred around stakeholder engagement, evidence, and paying attention to context • Developed for public health policy and practice • Fosters the creation of rapid learning organizations	• Relative lack of D&I constructs • No figure associated with this model
Dynamic Sustainability Framework (DSF) [47]	I	Framework centred around intervention, context, and the broader ecological system and their consideration over time Arose from the need to better understand how the sustainability of health interventions can be improved	• Focus on 5 socio-ecological levels: individual, organization, community, systems, and policy levels • Focus on continuous quality improvement to maximize programme fit, organizational learning, and stakeholder involvement • Developed for health services research	• Focus on sustainability Relative lack of specific D&I constructs • Framework needs to be refined and improved over time
Framework for enhancing the value of research for dissemination and implementation research [55]	D = I	Framework focused on the value of D&I research for end users and key D&I evaluation needs	• Focus on 5 socio-ecological levels: individual, organization, community, systems, and policy level • Focus on consistent evaluation including use of reporting and assessment tools • Focus on context, stakeholder engagement, and societal cost • Developed for public health	• Framework to advance D&I research in general aimed at informing D&I reporting guidelines • Lists essential domains but not D&I constructs • No figure associated with this model
The context and implementation of complex interventions (CICI) framework [48]		Framework for complex interventions focused on context, implementation, and setting interacting with each other and the intervention for use in systematic reviews and health technology assessments	• Solid conceptualization and development with step-by-step pragmatic guidance for operationalization • Applicable at micro, meso, and macro levels • Developed for public health	• Requires detailed assessment and reporting of primary research to populate the framework

Adapted from the overview by Tabak et al. [44] and a comparative analysis using the Dissemination-Implementation Webtool (https://dissemination-implementation.org/viewAll_di.aspx)

^#^D = Dissemination / I = Implementation. The focus is on dissemination and/or implementation activities. D—-only focuses on an active approach of spreading evidence-based interventions to target audience via determined channels using planned strategies. D = I, D > I, and I > D means there is some focus on both dissemination and implementation. I—only focuses on process of putting to use or integrating evidence-based interventions within a setting

**Fig. 2:**
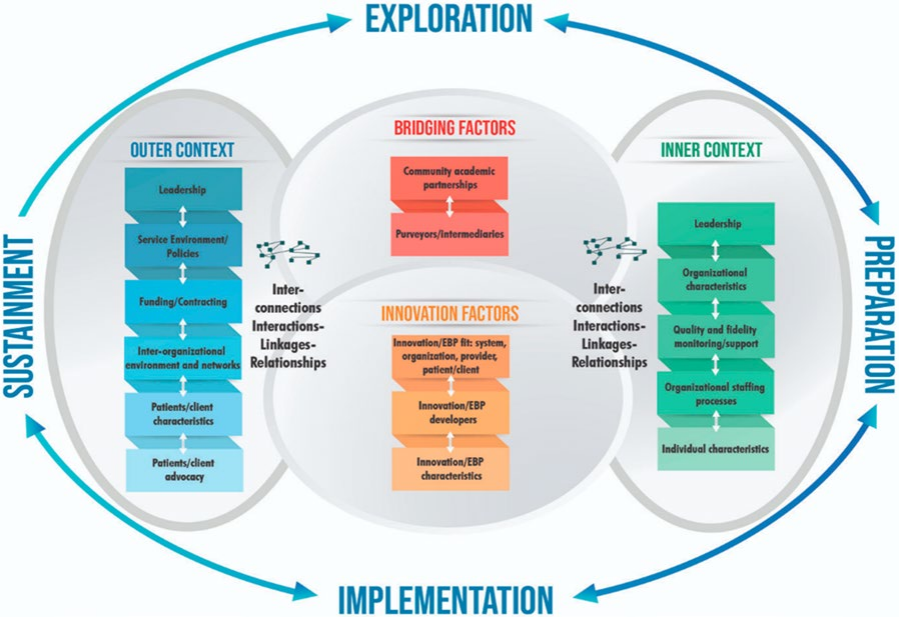
EPIS framework [40]

The EPIS framework, developed by Aarons et al*.* [40], describes four distinct phases to guide and describe the implementation process; enumerates factors within and across the inner and outer context across these phases; and outlines the factors that bridge the outer and inner context, as well as the interconnections and interlinkages that characterize the dynamics, complexity, and interplay of inner and outer contexts (Fig. 2). The EPIS framework was developed based on a literature review of implementation in public sector social and allied health service systems in the United States and found to be applicable to other countries and settings. However, until recently, the framework has shown limited prescriptive guidance for its use [56], and this paper aims to add evidence to the body of literature by focusing on its use in an upper-middle-income country.

The six different steps of the CEBHA+ IKT approach in South Africa were first mapped onto the different phases of the EPIS Framework (the sustainment phase was excluded given that the programme is still underway). The framework was then retrospectively applied to the process of IKT planning, implementation, and monitoring within CEBHA+ using a matrix analysis approach as described by Morse and Field [57]. Matrices allow mapping of relationships between as well as among constructs of interest. They also assist with exploring the relationship between constructs and theoretical concepts.

Several electronic data sources that were part of the South African planning and implementation folders were used to populate the EPIS matrix of constructs and phases. From the period since Step 1 (November 2018) until this reflection paper—Steps 5 and 6 (December 2020)—these sources include the South African CEBHA+ stakeholder analysis (November 2018), notes and minutes from eight quarterly South Africa CEBHA+ IKT team meetings, minutes from four consortium-wide meetings on IKT across all five countries, two presentations (2019 and 2020), and three scientific meeting presentations and posters (2020). We also consulted the individually tailored stakeholder engagement (or IKT) strategies for six stakeholders deemed to be priorities for the South African CEBHA+ team. Constructs were added or edited where appropriate or necessary, and analysis was continued through a series of online iterations until consensus on the mapping was reached by all authors.

## Results

**Fig. 3:**
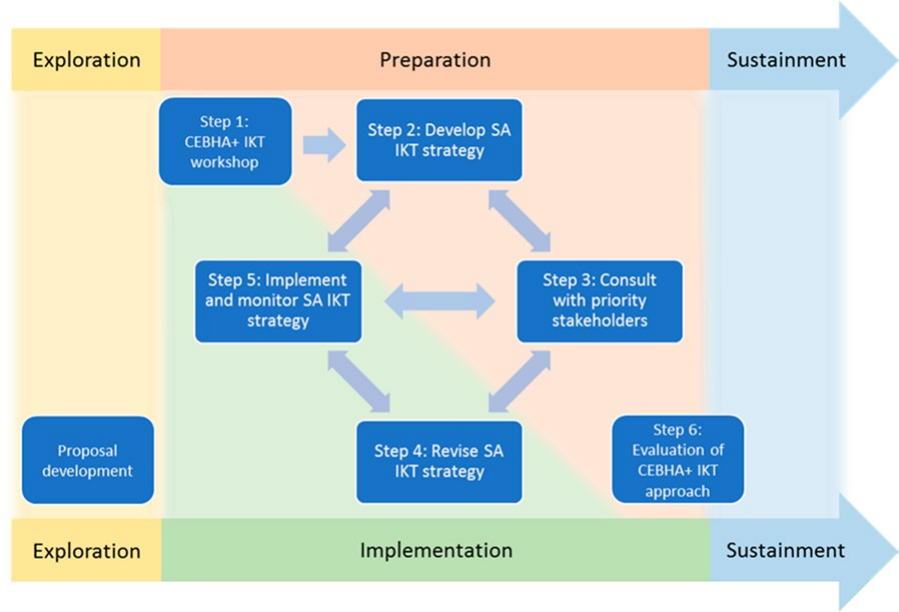
Revised South Africa CEBHA+ IKT approach

The mapping exercise revealed an IKT approach that was much more iterative, dynamic, and engaging than initially thought. The original plan (Fig. 1) evolved with several of the six steps overlapping across phases and interacting bidirectionally and with each other, as shown in Fig. 3.

**Fig. 4:**
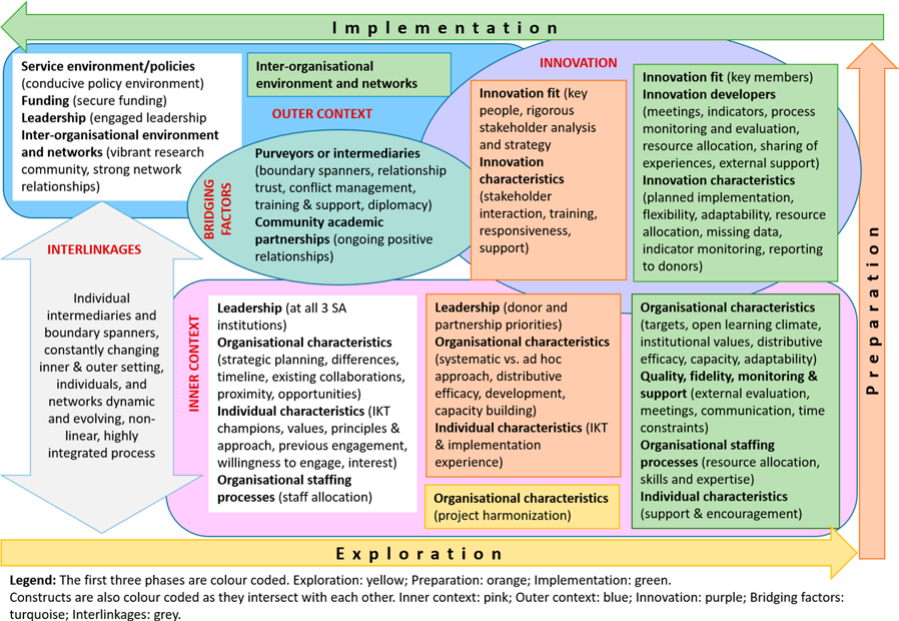
CEBHA+ IKT mapped onto EPIS

Several factors remained important and stable across phases (phase-agnostic), whereas others constantly evolved and adapted to the changing inner and outer contexts (phase-specific). Within each set of factors we discuss the EPIS subconstructs that are relevant to guide the interpretation of results. Other reflections and subconstructs not in the narrative can be found in Fig. 4 as well as Table 2, in which the constructs are embedded and colour-coded by phase. Factors that cut across phases remain in white, whilst those that are particular to a phase are shaded to match the colour of the relevant EPIS phase.

**Table 2:**
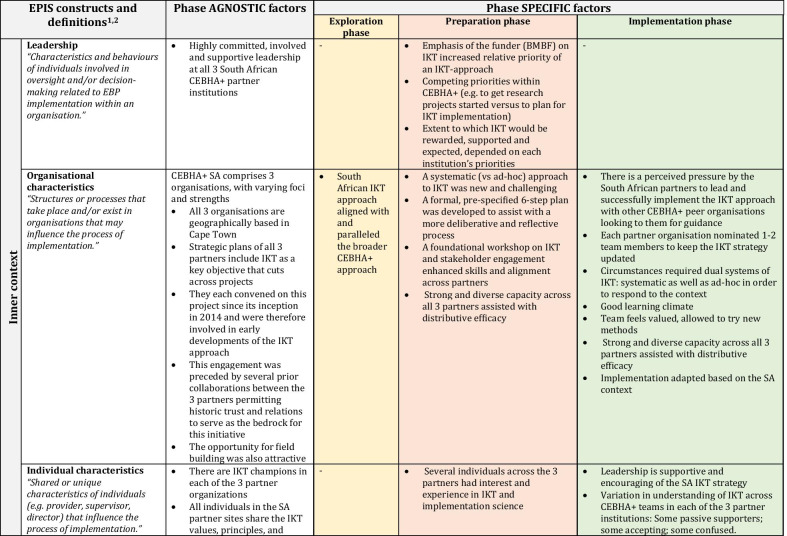

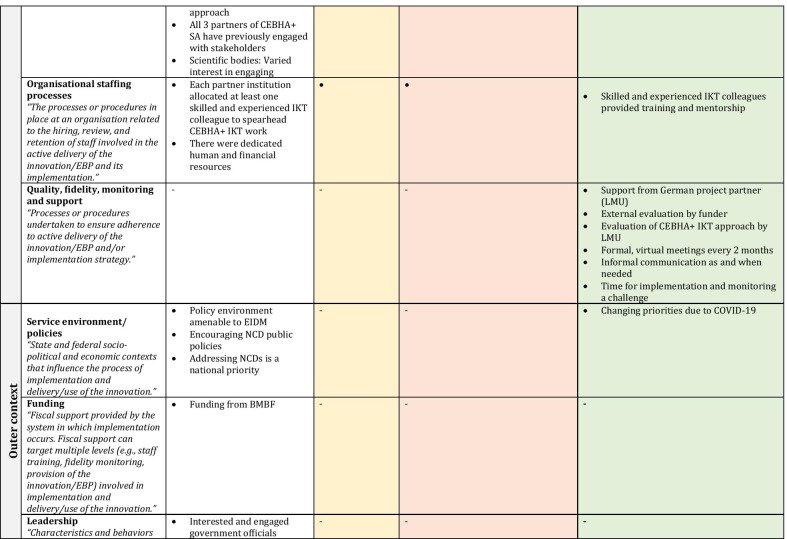

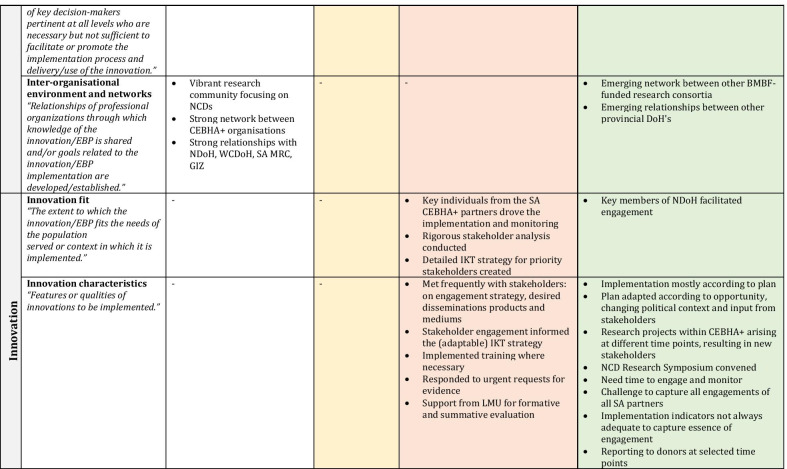

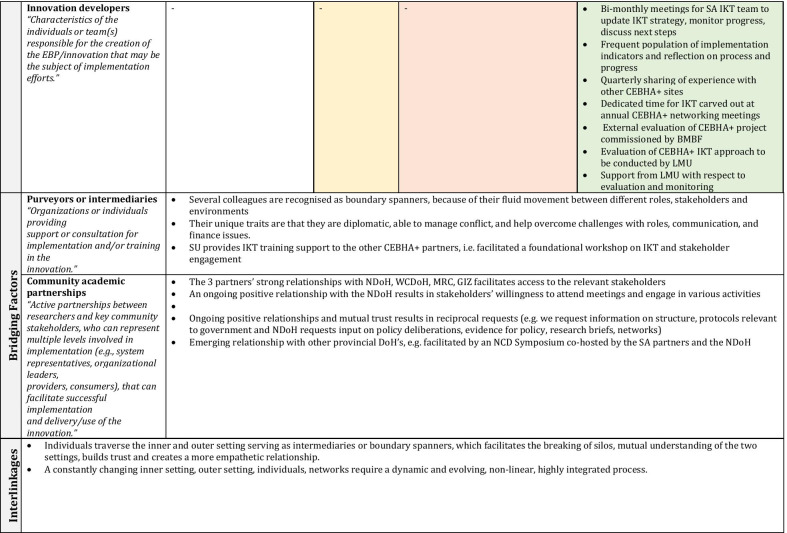
EPIS phases and constructs

Sources: Aarons et al. [40]; Becan et al. [34]

CEBHA+ : Collaboration for Evidence-based Healthcare and Public Health in Africa; SA: South Africa; IKT: Integrated knowledge translation; NdoH: National Department of Health; WCDoH: Western Cape Department of Health; MRC: Medical Research Council; GIZ: Deutsche Gesellschaft für Internationale Zusammenarbeit; LMU: Ludwig-Maximilian University of Munich; BMBF: Bundesministerium für Bildung und Forschung/Federal Ministry of Education and Research; EIDM: Evidence-informed decision-making; NCD: Non-communicable disease; COVID-19: Coronavirus Disease

### Phase-agnostic factors

#### Outer context

The idea to design and implement a comprehensive IKT approach as part of the CEBHA+ project was developed by CEBHA+ members during the exploration phase [37]. Although this was not a funding requirement or priority at that stage [58], our approach was very well received by the BMBF and led to an increased interest in and emphasis on IKT across the three phases reported on in this paper. Interest also grew amongst other BMBF-funded health networks during the preparation and implementation phases, allowing us to reflect on the interorganizational environment and network sub-constructs. For instance, during the implementation phase, interest in learning and adapting the approach developed by CEBHA+ resulted in an invited presentation and training workshop by CEBHA+ colleagues at the BMBF cross-network meeting in Ghana in January 2020.

Another pervasive factor across the three phases was the policy environment in South Africa that not only considered NCDs a national priority but also promoted EIDM. This enabled government officials to show interest in, and engage with, the CEBHA+ team and the vibrant South African research community in the field of NCDs.

#### Inner context

Highly committed, competent, and supportive leadership was found to be integral to the IKT approach across the three phases, particularly given its novelty to the programme and “learning by doing” design. Furthermore, at the individual level, some members of the CEBHC team that had worked across sectors and organizations with specific experience and knowledge of IKT served as IKT trainers for the whole CEBHA+ project. In addition, some government colleagues had a background in research and were therefore able to navigate the academic requirements associated with the CEBHA+ research projects.

This was enhanced by several organizational characteristics, including organizational structure and culture. For example, having all three institutions in Cape Town within proximity to each other permitted efficient communication, engagement, and co-leadership on several aspects of the CEBHA+ project but also IKT endeavours. Convening stakeholder meetings, embarking on joint approaches, and sharing of administrative, financial, and human resources was therefore easier. The fact that IKT was already implemented by the different partners as a pillar of their institutional vision also contributed to and facilitated stakeholder buy-in, championship, design, and integration of an IKT approach into the CEBHA+ project.

#### Bridging factors and interlinkages

Unique to the project was a recognition that several colleagues from the three partners as well as many stakeholders acted as intermediaries, or boundary spanners, who traversed easily between environments and roles, in turn providing a better appreciation and understanding of the complexities of the respective institutional structures and cultures. These boundary spanners facilitated the breaking of silos, enhancing mutual understanding between settings, building of trust, and creating a more empathetic relationship. Colleagues serving as intermediaries who are skilled in diplomacy and conflict management also helped to overcome challenges with roles, communication, and finance issues. The German coordinator, the funders, and technical support partners of CEBHA+ also played key bridging roles more internationally and across other health research networks.

Long-standing community–academic partnerships between individual team members at the three institutions with policy-makers at the provincial and national level facilitated access to many stakeholders. Furthermore, the fact that many colleagues from the National Department of Health (NDoH) had these positive ongoing relationships with members of the three institutions prior to the launch of the CEBHA+ project led to mutual trust, willingness to attend meetings, collaborate, and demand evidence from CEBHA+ partners to contribute to South African NCD policies and practices. As CEBHA+ research was rolled out and results and their implications shared, different stakeholders were involved at various times. For instance, the CEBHC recently convened stakeholders from the government (NDoH), research institutions (SAMRC), and civil society (The Cancer Association of South Africa) to discuss preliminary findings of a situation analysis of population-level interventions for diabetes and hypertension in South Africa. Similarly, multi-stakeholder engagement with management, health practitioners, and patients at two primary care clinics in Cape Town advanced individual counselling and enhanced group education for common NCDs, in turn leading to capacity strengthening of key partners.

### Phase-specific factors

#### Outer context

With respect to the interorganizational environment, we highlight that having all three institutions based in Cape Town was a notable strength with respect to a pre-existing relationship with the Western Cape DoH. However, this geographical concentration also proved to be a significant weakness, especially during the preparation phase, given the limited or absent relationships with other provincial DoHs. The implementation phase brought with it expanded networks including emerging relationships with other provincial DoHs, facilitated mainly through an NCD symposium co-hosted by the CEBHA+ partners in South Africa and the NDoH. This is particularly important given the decentralization of health services in South Africa, which lends executive decision-making power to different tiers of government.

In the implementation phase, each country, including South Africa, had to adapt the IKT approach to an evolving outer context. For instance, shifting priorities in the policy environment during the COVID-19 pandemic required swift pivots within the IKT process.

#### Inner context

While all three South African CEBHA+ partners had previously engaged with stakeholders, support by the funder in the preparation phase increased the relative priority of IKT and provided an opportunity for a more structured and explicit IKT design in the preparation and implementation phases. This led to a deliberate CEBHA+ South African IKT approach developed in line with the broader CEBHA+ programme across all African CEBHA+ partner countries during the preparation phase. During the early stage of implementation, organizational characteristics such as readiness for change and absorptive capacity were found to be important*.* Although IKT was a priority within CEBHA+ , the extent to which activities (that fell) within the remit of IKT would be rewarded, supported, and expected was relative to institutional priorities as well as readiness for change. Although the time for monitoring and implementing IKT in parallel with other CEBHA+ research activities proved challenging, the learning climate was very encouraging with the team feeling not only valued but also free to try new strategies and methods. In addition, the structured reflection allowed dedicated commitment from IKT team members as an organizational staffing strategy.

Finally, individual adopter characteristics were important, as instituting a systematic approach to IKT was new and proved challenging for many colleagues. Although individual attitudes were favourable, in the larger team there were some champions, some passive supporters, and some confusion. Due to varying experience in IKT and implementation science, some team members perceived a pressure to lead and successfully implement IKT in South Africa because peers were looking for guidance. For quality, fidelity, monitoring, and support purposes, virtual meetings were organized bimonthly during the implementation phase to monitor progress and adjust where necessary. This was complemented by informal communication, whether face to face or via email, as and when needed.

#### Innovation

As per Nilsen [42], innovation is the “implementation object”, which in this case was an embedded IKT approach*.* The ability of the IKT approach to be adaptable to a complex system that involved many stakeholders and evolving relationships was a key element of innovation characteristics. While the IKT approach and strategy was implemented mostly according to plan, it was adapted according to opportunity, changing political context, and input from stakeholders. This resulted in convening an NCD research symposium as a key activity in 2020 but also strengthened the need to monitor implementation indicators despite missing data being a challenge at times.

Innovation fit therefore was an important consideration. In the preparation phase, this included conducting a thorough stakeholder analysis, a stakeholder prioritization exercise, and design of a detailed IKT strategy for each priority stakeholder. Frequent meetings with key stakeholders permitted a better understanding of their engagement preferences, evidence needs, training requests, and output/product choices. A tailored engagement strategy and response was implemented based on this information, and adapted according to opportunities, changing political context, and input from stakeholders. At the implementation phase, this was supported by the NDoH.

Outcomes were captured systematically through a monitoring strategy maintained by IKT focal points across the three CEBHA+ partner institutions. Planned as well as opportunistic or demand-led engagements were captured in the IKT strategy documents in which the team reflected on process and progress, which was reported on as per donor requirements. This is particularly important for three reasons: (1) to adapt and evolve the IKT strategy as deemed necessary; (2) to share IKT experiences with other African CEBHA+ countries; and (3) to collect data relevant for external evaluation of the programme.

#### Bridging factors and interlinkages

Strong stakeholder relationships paid dividends during the implementation phase when key members of the NDoH facilitated engagement with other government stakeholders. For instance, when the South Africa CEBHA+ team hosted an NCD symposium in March 2020, we were able to rely on our social capital in the government to secure the attendance and contribution of the Minister of Health. Similarly, when decision-makers wanted to understand more about COVID-19 and its intersection with NCDs, South Africa CEBHA+ colleagues were considered key academics to be consulted for rapid evidence reviews [59, 60].

## Discussion

Since knowledge translation in global health started to gain traction in the lead-up towards the Millennium Development Goals [61, 62], efforts to bridge the implementation gap between knowledge production and EIDM in health policy have increased substantially. In low- and middle-income countries (LMICs), the marked inability of health systems to effectively implement evidence-informed interventions resulted in a call for implementation research in the design and execution of evidence-informed policy [63]. Although the relative paucity of peer-reviewed literature on IKT remains, there is an upward trend in documenting these experiences [64] as well as developing appropriate protocols [65]. Examples in Africa include the development of a KT platform in Zambia [66] and Malawi [67], and reflecting on KT strategies in Tunisia and Ivory Coast [35].

In this study, we documented our experience of developing, implementing, and monitoring the IKT approach in South Africa using the EPIS framework. As Becan et al. [34] pointed out, the EPIS model allows for examination of change processes at multiple levels, across time, and through successive stages toward implementation. The model was therefore well suited to understand and analyse our IKT approach. We combined the temporal aspect to demonstrate the dynamic and iterative IKT strategy that proved sensitive to changes in both the outer and inner context and was dependent on relationships and linkages between these. Using the EPIS framework to interrogate and document our IKT experiences proved extremely useful, particularly when mapping out the various phases as well as dynamic interactions that underpinned our IKT approach.

As outlined by Aarons et al*.* in their seminal paper [40], few implementation models explicitly recognize that different variables play crucially different roles at different time points during the process of implementation. In our study, several constructs remained stable across the three EPIS phases of exploration, preparation, and implementation, including stable and supportive funding, committed and competent leadership, skilled and dedicated IKT champions, diverse and established personal networks, a conducive and enabling policy environment, and boundary-spanning intermediaries. These phase-agnostic factors proved critical to ensure resilience and agility in the face of highly dynamic and changing local contexts and relationships, particularly as the COVID-19 pandemic hit. Some determinants of success were, however, phase-specific, which makes intuitive sense with respect to innovations. The IKT approach benefited from adaptation as the inner and outer context evolved. As a result, the team was able to respond to unanticipated demand-driven requests from decision-makers as well as pivot swiftly in response to crises. This agility will be important in the sustainment phase which we reflect later in the paper.

Reflections on the interlinkages and bridging constructs support the literature on the boundary spanners or knowledge brokers. Network analyses have often been used to unpack the role and power of such actors as both intermediaries as well as gatekeepers [26, 68–72]. While our experience with most stakeholders were positive in nature, several subconstructs within the inner context and outer context may shift this balance with a need for revised stakeholder analyses and relationship management [11, 73].

These findings will likely have implications for the sustainment phase where it will be important to capitalize on the phase-agnostic factors while managing for the phase-specific ones. Based on our experience and data, an institutionalization of IKT principles and practices such as authentic and mutually beneficial collaborations, a culture of EIDM, and capacity and resources for engagement and network maintenance will be critical. We propose that this can be imagined prospectively in our case as follows: (a) documenting the implementation of the IKT approach, specifically stakeholder engagement activities, will help us understand the changing needs and gaps in IKT or EIDM skills, and competencies amongst stakeholders; (b) maintaining collaborations as well as engaging with new stakeholders, particularly due to current and anticipated turnover within the government [11], potential internal organizational restructuring and/or staffing, and new actors in the external environment; (c) ensuring resilient institutional connections between CEBHA+ and stakeholders by going beyond single connections so as to have depth and breadth as well as diversity [26, 68]; (d) anticipating and planning for changing policy or decision-making processes; (e) continuously monitoring the implemented IKT approach to identify conversion or interchange between phase-agnostic and phase-specific factors across time, and whether newer strategies or innovations are required—this adaptation will be key to sustainment [56]; and (f) budgeting for cost-intense activities related to IKT. Longitudinal research of the CEBHA+ IKT strategy, as well as future initiatives such as the one described in this paper, would need to track some indicators to better understand sustainability and scalability which need more attention [56]. Such studies would help to understand the applicability of an IKT approach for projects and programmes beyond CEBHA+ that embed implementation science and IKT research into their design.

Having used the EPIS framework retrospectively, we were able to appreciate its value in prospective planning. This permits us to reflect on some lessons: had we used the framework earlier in the CEBHA+ project, we may well have considered what models best fit the IKT intervention in both our inner and outer context, keeping the diversity of our stakeholders as well as challenges—anticipated as well as unanticipated—in mind. The current IKT implementation and monitoring has relied on a handful of key individuals within the CEBHA+ team. More training of the rest of the team and perhaps even of stakeholders may have enhanced acceptability, fidelity, adaptation, and fit. Furthermore, as mentioned earlier, more deliberate thought to the sustainment of the value and benefit of the IKT approach would have likely occurred. With respect to capturing our dynamic programme, using a tool such as the framework for reporting adaptations and modifications (FRAME) [74] to capture change would perhaps have been helpful. This could be particularly important in approaching monitoring from a systems lens that needs to adjust for the complex nature of the IKT approach, which naturally introduces limitations to using any framework with inherent constraints and boundaries.

We acknowledge that our reflection has some limitations. Stakeholder engagement often happens on an ad hoc basis by CEBHA+ IKT team members and is not always formally captured. This may have resulted in missing data and limited analysis with room for more elaboration. Furthermore, documenting the process of stakeholder engagement also proved difficult in terms of how best to measure the success of engagement. Finally, research projects within CEBHA+ have started at various time points, resulting in new stakeholders being added to the IKT approach on a regular basis.

Given the reflexive nature of this paper, we recognize that the authors are all researchers on the CEBHA+ project and provide only one perspective of the IKT approach in South Africa. The paper is therefore limited to presentation of our own views and perspectives. However, the importance of stakeholder perspectives has been planned [38] and will be reported in a future publication that seeks to evaluate the CEBHA+ IKT approach across all African partner sites. We envisage that our perspective complements the broader evaluation, as it provides more in-depth contextualized information to help interpret the CEBHA+ IKT process and outcome results. Finally, we have not yet been able to reflect on the sustainment phase of the EPIS framework in our analysis given the ongoing implementation of the IKT approach.

## Conclusions

The challenge as well as opportunity in implementation science lies in the very nature of working in complex contexts with changing realities. Adapting the CEBHA+ IKT approach to respond to these changes was what permitted us to be agile, responsive, relevant, and useful to key decision-makers deliberating NCD policies and practices in a time of emergent crises. Bridging IKT with a framework from implementation science can be extremely beneficial not only when reflecting post facto on an IKT approach but also when planning and implementing such strategies. Documenting experiences from South Africa can contribute to strengthening the evidence base of such approaches from an LMIC perspective.

## Data Availability

The datasets used and/or analysed during the current study are available from the corresponding author on reasonable request.

## Abbreviations

BMBF: The Federal Ministry of Education and Research, Germany
CDIA: Chronic Disease Initiative for Africa
CEBHA+: Collaboration for Evidence-Based Healthcare in Africa
CEBHC, SU: Centre for Evidence-Based Health Care, Stellenbosch University
GIZ: Gesellschaft für Internationale Zusammenarbeit, Germany
IKT: Integrated knowledge translation
LMU: Ludwig Maximilian University of Munich
(N)DoH: (National) Department of Health
NCDs: Noncommunicable diseases
SAMRC: South African Medical Research Council
